# Genomic Landscape of Breast Cancer: Study Across Diverse Ethnic Groups

**DOI:** 10.3390/diseases13030086

**Published:** 2025-03-17

**Authors:** Asbiel Felipe Garibaldi-Ríos, Luis E. Figuera, Guillermo Moisés Zúñiga-González, Belinda Claudia Gómez-Meda, Ana María Puebla-Pérez, Alicia Rivera-Cameras, María Teresa Magaña-Torres, José Elías García-Ortíz, Ingrid Patricia Dávalos-Rodríguez, Mónica Alejandra Rosales-Reynoso, Patricia Montserrat García-Verdín, Irving Alejandro Carrillo-Dávila, Blanca Miriam Torres-Mendoza, Guadalupe Ávalos-Navarro, Martha Patricia Gallegos-Arreola

**Affiliations:** 1 División de Genética, Centro de Investigación Biomédica de Occidente, Centro Médico Nacional de Occidente, Instituto Mexicano del Seguro Social, Sierra Mojada #800, Guadalajara 44340, Jalisco, Mexico; asbiel.garibaldi4757@alumnos.udg.mx (A.F.G.-R.); luisfiguera@yahoo.com (L.E.F.); maganamt@gmail.com (M.T.M.-T.); jose.garciaor@imss.gob.mx (J.E.G.-O.); ingriddavalos@hotmail.com (I.P.D.-R.); patricia.garcia@alumnos.udg.mx (P.M.G.-V.); irving.carrillo4754@alumnos.udg.mx (I.A.C.-D.); 2 Doctorado en Genética Humana, Centro Universitario de Ciencias de la Salud, Universidad de Guadalajara, Guadalajara 44340, Jalisco, Mexico; 3 División de Medicina Molecular, Centro de Investigación Biomédica de Occidente, Centro Médico Nacional de Occidente, Instituto Mexicano del Seguro Social, Sierra Mojada #800, Col. Independencia, Guadalajara 44340, Jalisco, Mexico; mutagenesis95@hotmail.com (G.M.Z.-G.); mareynoso@hotmail.com (M.A.R.-R.); 4 Departamento de Biología Molecular y Genómica, Instituto de Genética Humana “Dr. Enrique Corona Rivera”, Centro Universitario de Ciencias de la Salud, Universidad de Guadalajara, Guadalajara 44340, Jalisco, Mexico; belinda.gomez@academicos.udg.mx; 5 Departamento de Farmacobiología, Centro Universitario de Ciencias Exactas e Ingenierías, Universidad de Guadalajara, Guadalajara 44430, Jalisco, Mexico; ana.puebla@academicos.udg.mx; 6 Departamento Ciclo de Vida, Genética y Medicina Genómica, Unidad Académica de Ciencias de la Salud, Universidad Autónoma de Guadalajara, Guadalajara 45129, Jalisco, Mexico; alicia.rivera@edu.uag.mx; 7 Laboratorio de Inmunodeficiencias Humanas y Retrovirus, División de Neurociencias, Centro de Investigación Biomédica de Occidente, Centro Médico Nacional de Occidente, Instituto Mexicano del Seguro Social, Guadalajara 44340, Jalisco, Mexico; blanca.torresm@imss.gob.mx; 8 Departamento de Disciplinas Filosófico Metodológicas, Centro Universitario de Ciencias de la Salud, Universidad de Guadalajara, Guadalajara 44340, Jalisco, Mexico; 9 Departamento de Ciencias Médicas y de la Vida, Centro Universitario de la Ciénega, Universidad de Guadalajara, Av. Universidad 1115, Lindavista, Ocotlán 47820, Jalisco, Mexico; guadalupe.avalos5337@academicos.udg.mx

**Keywords:** breast neoplasms, cancer genomics, ethnic groups, genetic variation, mutation, computational biology, cancer hallmarks, genomic variation in cancer, comparative cancer genomics, molecular cancer biology

## Abstract

**Background**: Breast cancer (BC) is the most common cancer among women worldwide, with incidence and mortality rates varying across ethnic groups due to sociodemographic, clinicopathological, and genomic differences. This study aimed to characterize the genomic landscape of BC in diverse ethnic groups using computational tools to explore these variations. **Methodology**: cBioPortal was used to analyze genomic, clinicopathological, and sociodemographic data from 1084 BC samples. Mutated genes were classified based on GeneCards platform data. Enrichment analysis was performed with CancerHallmarks, and genes not found were compared with MSigDB’s Hallmark Gene Sets. Genes absent from both were further analyzed using NDEx through Cytoscape.org to explore their role in cancer. **Results**: Significant differences (*p* < 0.05) were observed in sex, tumor subtypes, genetic ancestry, median of the fraction of the altered genome, mutation count, and mutation frequencies of genes across ethnic groups. We identified the most frequently mutated genes. Some of these genes were found to be associated with classic cancer hallmarks, such as replicative immortality, sustained proliferative signaling, and the evasion of growth suppressors. However, the exact role of some of these genes in cancer remains unclear, highlighting the need for further research to better understand their involvement in tumor biology. **Conclusions**: This study identified significant clinicopathological and genomic variations in BC across ethnic groups. While key genes associated with cancer hallmarks were found, the incomplete characterization of some highlights the need for further research, especially focusing on ethnic groups, to understand their role in tumor biology and improve personalized treatments.

## 1. Background

Breast cancer (BC) is a genetic and multifactorial disease and one of the leading causes of female mortality worldwide, making it a worrying public health problem [1,2,3]. Despite ongoing advances in our understanding of BC, its genomic landscape remains highly heterogeneous, with mutations detected across a broad range of genes [4,5]. These mutations can promote the carcinogenic process in the breast and even influence the clinicopathological features of the disease [6,7]. Therefore, the biological and clinical heterogeneity of BC promoted by its genetic heterogeneity remains a challenge.

BC genomics play a pivotal role in understanding the mechanisms of the disease [4,8]. Genomic studies are essential for identifying mutations that may influence key aspects of the disease, such as susceptibility, aggressiveness, and response to treatment [9,10,11]. The identification of these mutations has a direct impact on improving diagnostic and therapeutic precision and on advancing personalized treatment strategies [12].

It is to be expected that the genomic heterogeneity of BC also differs between ethnic groups, as it has been shown that different ethnic groups have unique genetic profiles that may influence the presentation of the disease [13,14,15]. For this reason, understanding these genomic differences may help in the identification of risk factors, new therapeutic targets, and more effective personalized treatments.

This study conducts a computational analysis of the genomic landscape of BC, with the aim of identifying and describing the most frequently mutated genes, as well as the types of mutations present in different ethnic groups. Through this analysis, a precise view of commonly mutated genes and their prevalence across distinct ethnic groups is provided, along with a comparison of the frequencies of the most mutated genes in the categories of structural variants (SVs), copy number alterations (CNAs), and point mutations across the studied ethnic groups. Additionally, clinicopathological differences associated with the disease were evaluated, such as the distribution of sex, age, tumor type, and subtype, which are key factors for understanding the clinical heterogeneity of BC. Genomic aspects, such as mutation count and the fraction of genome altered (FGA), were also considered, as they are essential indicators for assessing the genomic burden of tumors. This study further explores how the genes with alterations observed in different ethnic groups are associated with key and distinctive features of cancer, such as the ability to evade the immune system, the sustainability of cellular proliferation, and other hallmark characteristics of cancer. This approach provides valuable information that could guide future research and the development of personalized therapeutic strategies, considering both the genetic and ancestral characteristics of patients.

## 2. Materials and Methods

### 2.1. Study Samples

This study was conducted using data extracted from the cBioPortal platform [16] https://www.cbioportal.org, accessed on 4 March 2025). The cBioPortal platform is a public repository that allows for the visualization, analysis, and interpretation of cancer genomic data obtained from large-scale genetic and genomic studies, including data from The Cancer Genome Atlas (TCGA) and PanCancer Atlas. TCGA [17] is a large-scale megaproject aimed at characterizing the molecular aspects of more than 33 types of cancer. This project collected genomic, transcriptomic, proteomic, and clinical data from over 20,000 primary cancer samples along with their corresponding normal samples. As a result, it provided highly relevant data, such as mutations and gene expression levels. On the other hand, the PanCancer Atlas [18] focused on comparing the 33 cancer types characterized by TCGA to identify common patterns among them. This approach has provided a more comprehensive view of cancer biology and has facilitated the discovery of therapies that, while effective for one type of tumor, could also be applicable to others with a similar genomic profile.

For this study, we selected the “Breast Invasive Carcinoma” dataset from TCGA [17,19] and the PanCancer Atlas [18], which includes 1084 samples of BC. The samples were classified according to their reported ethnic background in the Breast Invasive Carcinoma study: White (*n* = 651), Black or African American (*n* = 166), Asian (*n* = 57), Hispanic or Latino (*n* = 33), and American Indian/Alaska Native (*n* = 1).

Given that there is only a single representative sample for the American Indian/Alaska Native group, it was excluded from further analyses. However, relevant findings from this sample will be mentioned throughout the Results and Discussion Sections where appropriate, to ensure its potential implications are acknowledged.

The dataset included clinical, sociodemographic, and ancestral information, such as ethnic origin, genetic ancestry, and sex, as well as tumor type and subtype. Additionally, it provided the genomic characteristics detailed in this study.

### 2.2. Genomic Characteristics of BC by Ethnic Group

The genomic characterization of BC by ethnic group was obtained from the dataset provided by cBioPortal [16], including the Fraction of Genome Altered (FGA) to assess the extent of genomic alterations. Genes with structural variants (SVs), copy number alterations (CNAs), and point mutations were directly provided by cBioPortal [16], including their frequencies and percentages.

For filtering mutated genes, the following criteria were applied to each ethnicity: the top 3 genes with the highest number of SVs present in at least two samples were selected and in the case of ties (i.e., genes with the same number of SVs), all tied genes were included in the top 3. Similarly, the top 3 genes with the most frequent CNAs were selected, and genes with the same frequency were also included in the top 3. Additionally, genes with point mutations present in more than 10% of the samples were considered in the filtering process. In cases where multiple genes had the same number of mutations, all were included to ensure the most relevant genes were considered. This process was applied to each ethnicity individually.

### 2.3. Comparison of the Most Frequently Mutated Genes Across Ethnic Groups

After applying our filtering criteria, we identified the most frequently mutated genes in each ethnic group for SVs, CNAs, and point mutations. The mutation frequency of these genes was then compared across all ethnic groups to assess significant differences. In cases where multiple genes had the same mutation frequency within an ethnic group, all tied genes were included in the analysis.

### 2.4. Comparison of Altered and Non-Altered Groups Based on Mutation Status

Once the filtering criteria were applied to the genes for each ethnicity, we grouped the samples into two categories based on the mutations present in the filtered genes. The “altered group” included samples with mutations in the genes that passed our filtering criteria. The “non-altered group” consisted of samples without mutations in these filtered genes, but which still contained mutations in genes that were excluded from the filtering criteria. These groups were then compared with the clinical and pathological characteristics of the disease.

### 2.5. Gene Classification

Once the most frequently mutated genes in BC for each ethnicity were filtered, they were combined into a single set and manually classified using information obtained from the GeneCards database [20] (https://www.genecards.org, accessed on 4 March 2025).

GeneCards [20] is a free, online platform and database that provides detailed information about human genes sourced from experimental data and publications. This platform facilitates access to key data in human genetics, allowing users to explore the essential characteristics of genes, such as their functions, expression patterns, interactions in biological processes, and even their associations with various diseases.

The combined set of genes was categorized into the following classes based on the data obtained from GeneCards [20]: oncogenes, tumor suppressors, transcription factors and regulators, structural proteins, kinases and enzymes, receptors and ion channels, metabolic regulators and transporters, signaling molecules, and others.

### 2.6. Enrichment Analysis

Additionally, an enrichment analysis was performed on the CancerHallmarks platform [21] (https://cancerhallmarks.com, accessed on 5 March 2025) using the combined set of the most frequently mutated genes in BC across all ethnic groups. This platform integrates over 6700 genes identified or involved in the fundamental biological processes of cancer, such as cell proliferation, evasion of cell death, metastasis, and more. In this way, it groups genes based on their biological functions, facilitating the interpretation of their role in tumor progression.

CancerHallmark [21] functionally provides two gene sets to compare with our genes of interest: the “Integrated Cancer Hallmark Gene Set” and the “Core Cancer Hallmark Gene Set”. The first set comprises 6763 individual genes, created by combining multiple sources, with most genes derived from a single reference. In contrast, the “Core Cancer Hallmark Gene Set”, consisting of 1574 genes, includes those that appear in at least two references, suggesting stronger scientific evidence for their role in cancer biology. These sets were used to perform an enrichment analysis with our gene sets of interest, allowing us to identify overrepresented biological processes or pathways associated with cancer. Enrichment analysis is a method used to determine whether a specific set of genes is significantly associated with particular biological processes or pathways. In this context, it helps identify whether certain cancer-related processes or pathways are more frequently represented in the gene sets compared to what would be expected by chance.

The filtered genes not associated with specific hallmarks in the CancerHallmark platform were compared with the “Hallmark Gene Sets” obtained from the Molecular Signatures Database (MSigDB) [22] (https://www.gsea-msigdb.org/gsea/index.jsp, accessed on 5 March 2025), hosted by the Broad Institute. The comparison was made to check if the genes not appearing in the CancerHallmark platform were present in the MSigDB set. The MSigDB “Hallmark Gene Sets” [22] group genes into coherent functional signatures, representing well-defined biological processes, such as cell proliferation, angiogenesis, inflammatory response, and others. This comparison helped identify key patterns associated with cancer characteristics.

As a final filter, genes not identified on the previous platforms were analyzed using NDEx [23], accessed through Cytoscape.org [24], in the “Analyze Your Genes With NDEx iQuery” section (https://cytoscape.org, accessed on 5 March 2025), to determine if they belong to any relevant molecular pathways in cancer biology. NDEx [23] is a platform that facilitates the sharing, visualization, and analysis of biological networks, integrated with Cytoscape [24] to represent gene interactions within well-defined biological pathways. While neither platform is specifically designed for enrichment analysis, their utility in this study lies in their ability to visualize genes not included in cancer-associated gene sets from previous platforms, enabling us to explore how these genes may influence cancer biology based on the biological pathways in which they are involved.

### 2.7. Statistical Analysis

To compare clinical, sociodemographic, and ancestral characteristics between ethnic groups, as well as mutational patterns in altered and non-altered groups, Chi-square tests were used for categorical variables, including proportions of FGA, mutation counts, and the frequency of the most frequently mutated genes in each ethnicity. These frequencies were then compared with those in other ethnic groups. The Kruskal–Wallis test was applied to compare the medians of continuous variables, such as mutation count and FGA. *p*-values were calculated to determine significance, with values of less than 0.05 considered significant. Subsequently, these *p*-values were adjusted to control the false discovery rate using *q*-values, with values of less than 0.05 considered significant.

Due to the presence of only a single sample in the American Native/Alaska Native group, this ethnicity was excluded from the statistical analysis, as meaningful comparisons could not be performed.

For enrichment analysis, values with *p* < 0.05 and *p*.adj < 0.05 were considered statistically significant.

## 3. Results

### 3.1. Sociodemographic Characteristics

**Figure 1:**
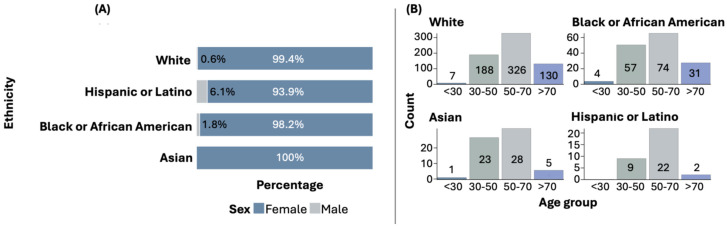
Sex (**A**) and age (**B**) distribution by ethnicity.

Among all ethnic groups, females are predominantly affected by BC. However, in the Hispanic or Latino group, the proportion of males with BC is higher compared to other ethnic groups. Statistically significant differences were found when comparing the sex distribution across ethnic groups (*p* = 0.0104). Figure 1A illustrates the distribution of sex by ethnic group.

### 3.2. Clinicopathological Characteristics

The most common age at diagnosis is above 50 years, although early diagnoses (under 30 years and between 30 and 50 years) are also observed in some ethnic groups. Figure 1B shows the distribution of age at diagnosis by ethnic group. The distribution of age at diagnosis among the ethnic groups did not show statistically significant differences.

In terms of tumor type and subtype, infiltrating ductal carcinoma is the most common type across all ethnic groups. Regarding tumor subtype, Luminal A is the most prevalent in most ethnic groups (Table 1). When comparing the frequencies of tumor types, no statistically significant differences were observed among the ethnic groups. However, significant differences were found when comparing the ethnic groups based on the frequencies of tumor subtypes (*p* < 0.0001). The single American Indian/Alaska Native sample was classified as infiltrating ductal carcinoma, subtype Her2.

### 3.3. Genetic Ancestry

**Figure 2:**
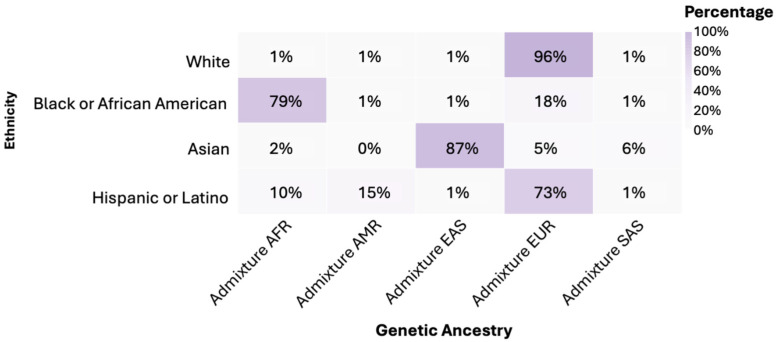
Genetic ancestry by ethnicity. Notes: AFR: African; AMR: Native American; EAS: Eastern Asian; EUR: European; SAS: South Asian.

It was found that 96% of individuals in the White group had predominantly European (EUR) genetic ancestry. In the Black or African American group, 79% had African (AFR) genetic ancestry. Asian showed an 87% predominance of East Asian (EAS) genetic ancestry. Hispanic or Latino exhibited a 73% European (EUR) genetic admixture (Figure 2). The American Indian/Alaska Native group was classified within its own ethnic group. When comparing genetic ancestries among White, Black or African American, Asian, and Hispanic or Latino ethnicities, statistically significant differences were observed in the distribution of frequencies (*p* < 0.0001).

### 3.4. Genomic Characteristics of BC

**Figure 3:**
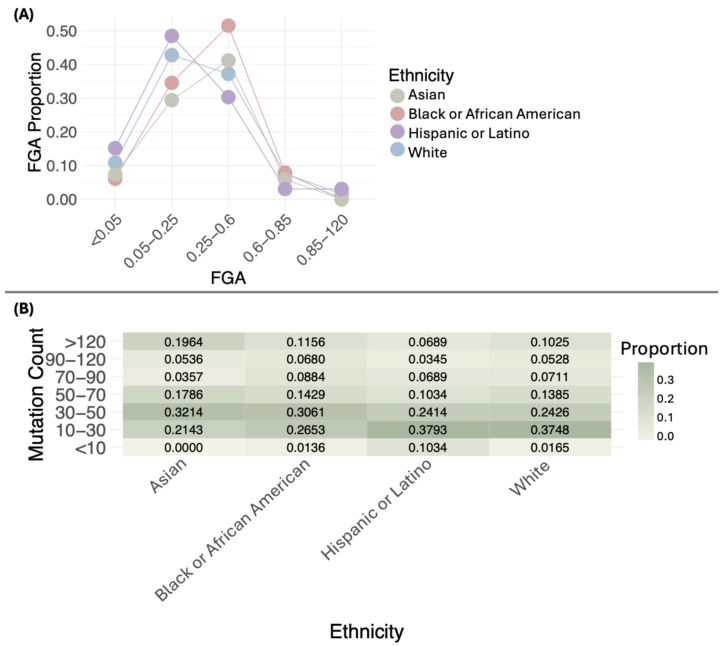
Distribution of the proportion of the fraction of genome altered (FGA) (**A**) and mutation count (**B**) by ethnicity.

The distribution of FGA proportions across different ethnic groups shows distinct patterns. Most White, Black or African American, Asian, and Hispanic or Latino samples fall within the 0.05–0.6 range, with varying proportions. Values below 0.05 and above 0.6 are uncommon in all groups. No significant differences were observed in the distribution of FGA proportions across ethnicities (*p* = 0.05484). Figure 3A illustrates these distributions, while Table S1 provides detailed sample counts for each FGA interval by ethnicity. The single Native American/Alaska Native sample has an FGA of 0.19 in the 0.05–0.25 interval.

When comparing the medians of FGA across ethnic groups, the following values were observed: White (0.24), Black or African American (0.32), Asian (0.30), and Hispanic or Latino (0.19). Statistically significant differences were found in the medians of FGA (*p* = 0.006941) (Table S2), with the lowest median in the Hispanic or Latino group.

The distribution of mutation proportions across different ethnic groups shows distinct patterns. Most White samples are concentrated in the 10–30 range, with a proportion of 0.3748, followed by the 30–50 range, with a proportion of 0.2426. A notable proportion is also found in the >120 category, with 0.1025. Black or African American samples generally show lower mutation proportions, with most cases in the 10–30 range (0.2653) and 30–50 range (0.3061), and fewer samples exceeding 120 mutations (0.1156). Asian samples have a concentration of mutations in the 30–50 range (0.3214) and a smaller proportion in the >120 category (0.1964). Hispanic or Latino samples primarily exhibit mutations in the 10–30 range (0.3793), with only a small proportion surpassing 120 mutations (0.0689). The single Native American/Alaska Native sample has a mutation count of 24. When comparing the distribution of mutation count proportions, no statistically significant differences were observed (*p* = 0.2232).

Statistically significant differences were found in mutation counts among ethnic groups (*p* = 0.001801) (Figure 3B, Table S1). Asian and Black or African American had the highest medians, with values of 46 and 43, respectively, followed by White (37) and Hispanic or Latino (31) (Figure 3B, Table S1).

### 3.5. Most Frequently Mutated Genes in BC

Before applying the filtering criteria, in the White ethnicity, there were 14,796 genes with point mutations, 3831 genes with SVs, and 34,108 genes with CNAs. In the Black or African American group, 1564 genes had SVs, 22,766 genes had CNAs, and 5886 genes had point mutations. For the Asian group, 729 genes had SVs, 1530 genes had CNAs, and 3441 genes had point mutations. The Hispanic or Latino group showed 263 genes with SVs, 7116 genes with CNAs, and 3849 genes with point mutations. Finally, the American Native/Alaska Native sample had 28 genes with SVs, 255 genes with CNAs, and 25 genes with point mutations.

After applying the filtering criteria, the most frequently mutated genes, including those with SVs, CNAs, and point mutations across different ethnicities, were identified as follows: 19 genes in White; 20 genes in Black or African American; 35 genes in Asian; 19 genes in Hispanic or Latino; 11 genes in American Native/Alaska Native.

**Figure 4:**
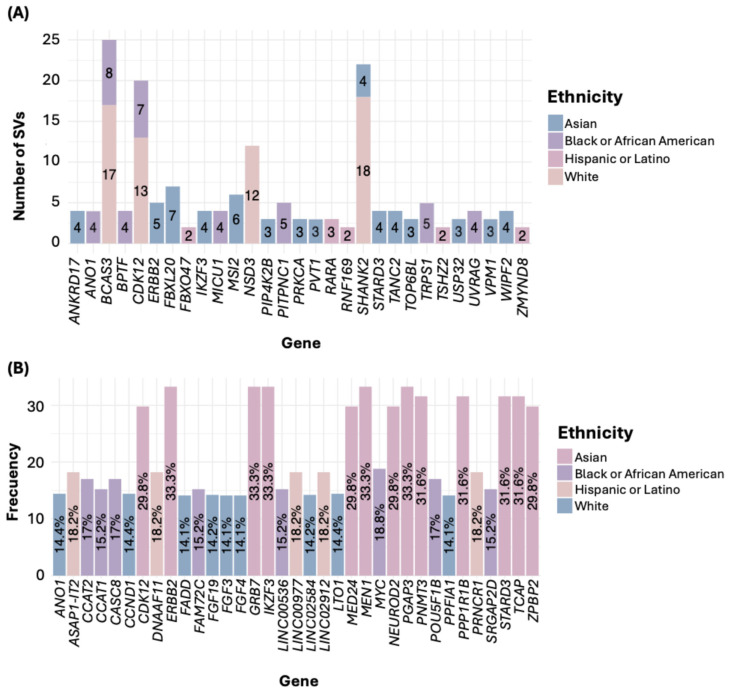
Number of SVs (**A**) and CNAs (**B**) by ethnicity.

Regarding SVs, in the White ethnicity, the gene *SHANK2* exhibited the highest number of SVs. For the Black or African American group, the most affected gene was *BCAS3*; in the Asian group, it was *FBXL20*; in the Hispanic or Latino group, it was *RARA* (Figure 4A). Notably, all filtered SVs corresponded to gene fusions (Table S3). In the American Native/Alaska Native group, we identified genes such as *DENND1A* and *PCYT2*, each with two SNVs.

Concerning genetic alterations related to CNAs, all were gene amplifications (Table S4). In the White group, the genes *LTO1*, *ANO1*, and *CCND1* showed the highest frequency of amplifications. In the Black or African American group, the gene *MYC* was the most frequently amplified. Among the Asian group, the most common amplifications were in the genes *PGAP3*, *MIEN1*, *ERBB2*, *IKZF3*, and *GRB7*. In the Hispanic or Latino group, frequent amplifications were observed in the genes *PRNCR1*, *LINC02912*, *LINC00977*, *ASAP1-IT2*, and *DNAAF11* (Figure 4B, Table S4). In the American Indian/Alaska Native sample, the genes *RBIS*, *SECTM1*, and *CXCL5* were the most frequently amplified.

**Figure 5:**
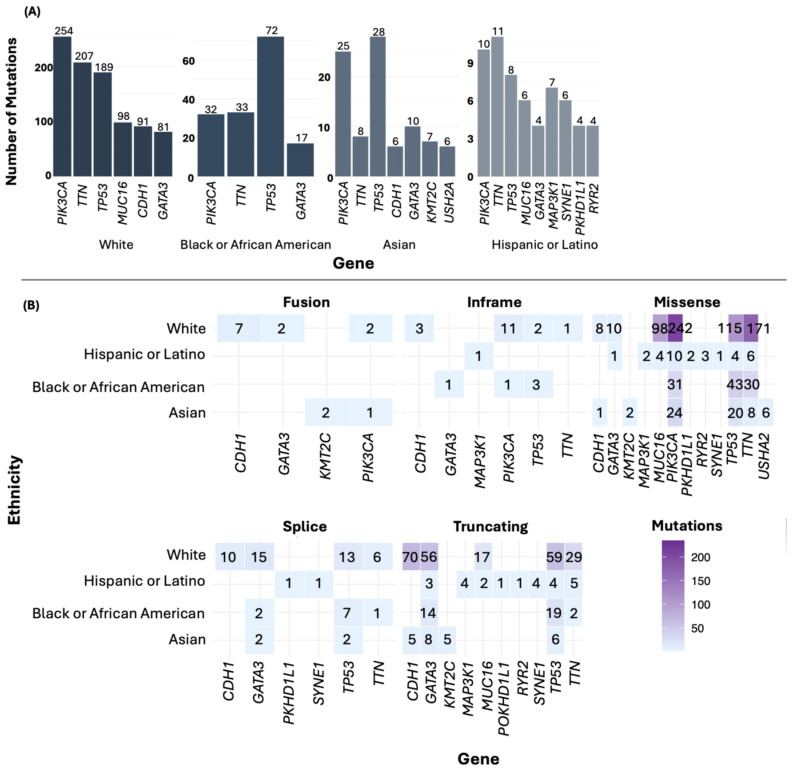
Point mutations (**A**) and type of mutations by ethnicity (**B**).

For the most frequently mutated genes in BC by ethnicity, *PIK3CA* was the most mutated gene in the White group. In the Black or African American and Asian groups, *TP53* was the most mutated gene, while in the Hispanic or Latino group, *TTN* was the predominant gene (Figure 5A). Additionally, among the most frequently mutated genes, missense mutations were the most common (Figure 5B). In the American Indian/Alaska Native sample, mutations were found in numerous genes, including *MLXIP*, *DOCK4*, *EPHA6*, *CHRNA5*, and *INS.*

### 3.6. Frequency of the Most Frequently Mutated Gene Across Ethnic Groups

The most frequently mutated genes in each category (SVs, CNAs, and point mutations) were compared across all ethnic groups to assess whether their mutation frequencies differed significantly among the ethnic groups.

In the comparison of genes mutated by SVs, *ERBB2* showed a significantly higher frequency in the Asian group (8.77%, *p* < 0.0001). *FBXL20* was also more frequent in the Asian group (8.77%, *p* = 0.0039), as well as *STARD3* (7.02%, *p* = 0.000343) and *IKZF3* (7.02%, *p* = 0.002723). In the Hispanic or Latino group, the genes *FBXO47* (6.06%, *p* < 0.0001), *ZMYND8* (6.06%, *p* < 0.0001), and *RNF169* (6.06%, *p* = 0.000859) showed higher frequencies. Additionally, *TSHZ2* was found higher in the Hispanic group (6.06%, *p* = 0.00122) (Table 2).

Regarding the comparison of genes with CNAs, *PG*AP3, *MIEN1*, *ERBB2*, *IKZF3*, and *GRB7* had a frequency of 33.33% in the Asian group (*p* < 0.0001), while in the White and Black or African American groups, the frequency ranged from 9.29% to 11.52%, and in the Hispanic or Latino group, it was 6.06%. Similarly, *TCAP*, *PNMT*, *STARD3*, and *PPP1R1B* showed alterations in 31.58% of the samples in the Asian group, whereas their frequency in the Hispanic or Latino group was 6.06% (*p* < 0.0001). Other genes with high CNA frequencies in the Asian group included *MED24*, *NEUROD2*, *ZPBP2*, and *CDK12*, all with a prevalence of 29.82%, compared to lower frequencies in the White, Black or African American, and Hispanic or Latino groups (*p* < 0.0001) (Table 2).

Regarding point mutations, *TP53* showed a lower frequency in the Hispanic or Latino group (21.21%) compared to White (29.22%), Black or African American (43.21%), and Asian (49.12%, *p* = 0.000127). On the other hand, *PIK3CA* showed a lower frequency in the Black or African American group (19.14%) compared to White (35%), Asian (38.60%), and Hispanic or Latino (27.27%, *p* = 0.000926) (Table 2).

### 3.7. Comparison of Altered and Non-Altered Groups

In the White group, statistically significant differences were found in the distribution of tumor type and subtype when comparing the altered group with the non-altered group (*p* < 0.0001 and *p* < 0.001464, respectively).

For the Black or African American group, significant differences were observed in tumor subtype between the altered and non-altered groups (*p* = 0.0006288).

In the Asian group, significant differences were detected in both tumor type (*p* = 0.002541) and subtype (*p* < 0.0001).

In the Hispanic or Latino group, no significant differences were observed in the clinical or pathological parameters.

### 3.8. Classification of Mutated Genes in BC

Considering all ethnic groups, and after applying our filtering criteria, a set of 71 genes was created, identified as mutated in BC through point mutations, SVs, or CNAs. The most frequently mutated genes fall into the category of kinases and enzymes, followed by oncogenes, and thirdly, the category of others. The “others” category includes non-coding RNAs, pseudogenes, secreted and transmembrane proteins, components of ubiquitin ligases, and apoptosis regulators (Table 3).

### 3.9. Identification of Enriched Cancer Hallmarks

In the analysis conducted on the CancerHallmarks platform [21], based on the “Integrated Cancer Hallmarks Gene Set”, genes associated with the following hallmarks were identified: seven in “Sustained Angiogenesis”, six in “Tumor-Promoting Inflammation”, ten in “Genome Instability”, twenty-two in “Sustaining Proliferative Signaling”, seven in “Evading Immune Destruction”, nine in “Replicative Immortality”, fifteen in “Resisting Cell Death, twenty in “Evading Growth Suppressors”, eight in “Reprogramming Energy Metabolism”, and eighteen in “Tissue Invasion and Metastasis” (Table 4).

**Figure 6:**
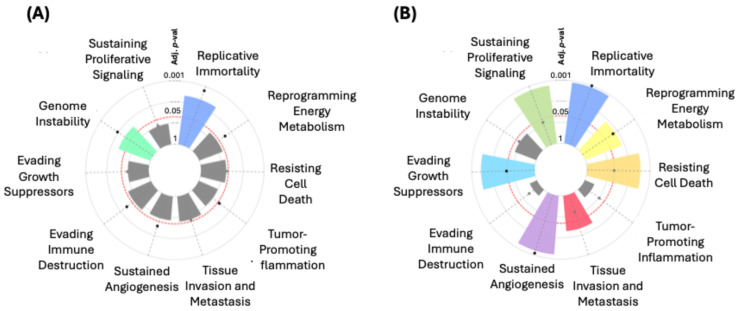
Hallmark enrichment analysis for the “Integrated Cancer Hallmark Gene Set” (**A**) and the “Core Cancer Hallmark Gene Set” (**B**).

In the hallmark enrichment analysis, statistically significant results were observed (Figure 6A) for “Replicative Immortality” and “Genome Instability” for the “Integrated Cancer Hallmark Gene Set” (*p* = 0.00636 and *p* = 0.03106, respectively). On the other hand, for the “Core Cancer Hallmark Gene Set”, significant findings were noted in “Replicative Immortality” (*p* < 0.0001) and “Sustained Angiogenesis” (*p* = 0.00040). Significant associations were also identified for “Evading Growth Suppressors” (*p* = 0.00144) and “Resisting Cell Death” (*p* = 0.00184). Other terms such as “Tissue Invasion and Metastasis” (*p* = 0.01793) and “Reprogramming Energy Metabolism” (*p* = 0.00945) also showed significance (Figure 6B).

Additionally, genes not initially associated with specific hallmarks on the CancerHallmark platform were compared with the MSigDB “Hallmark Gene Sets” [22]. This comparison allowed us to assess whether these genes were part of the MSigDB hallmarks. For genes not identified in the previous platforms, we also explored their involvement in molecular pathways using NDEx [23], accessed through Cytoscape.org [24]. This analysis provided insights into the potential pathways these genes might be associated with in cancer biology.

Based on these findings, we manually proposed hallmark classifications for these genes, which were not initially assigned to any hallmark. Table 5 presents the genes based on the MSigDB “Hallmark Gene Sets” [22] along with their proposed integration into cancer hallmarks.

Genes not linked to any hallmark in previous analyses were further explored using NDEx [23] through Cytoscape.org [24]. Table 6 lists the identified genes along with their proposed integration into cancer hallmarks.

## 4. Discussion

Our results regarding sociodemographic and clinicopathological characteristics align with the observation that BC predominantly affects women across all ethnic groups [25]. However, we observed a higher proportion of men with BC in the Hispanic or Latino group compared to other ethnic groups. Although no significant differences were found in the age at diagnosis among the groups, our finding that most cases occur in women over 50 years old is consistent with previous studies [26,27], which also observed this trend across multiple ethnic groups.

Regarding tumor subtypes, we found significant differences in the frequencies of subtypes among ethnicities, with Luminal A being the most common. This is consistent with a previous study [28], which also observed a higher prevalence of luminal subtypes but with ethnic variations in their proportions. These differences underscore the importance of continuing to investigate the molecular characteristics of BC in diverse ethnic groups to better understand the biological and sociodemographic factors influencing its development and clinical behavior.

In our study, we analyzed the genetic ancestry of different ethnic groups, revealing significant differences across them. The White group predominantly exhibited European ancestry, aligning with findings from other studies that highlight the complex European origin of this ethnic group due to immigration patterns from various European regions [29]. In contrast, 79% of the African American group showed strong African ancestry, reflecting their complex genetic history, and consistent with other research emphasizing the significant African contribution within this group [30]. The Asian samples were highly homogeneous, with 87% presenting East Asian ancestry, in line with previous studies identifying East Asian ancestry as dominant within this ethnic group [31]. Hispanic or Latino displayed a 73% European genetic admixture, highlighting the historical blending involving Indigenous, European, and, to a lesser extent, African ancestry. Other studies also underscore the genetic diversity within the Latino ethnicity due to the mixing of different ancestries, reinforcing the importance of considering this genetic complexity when analyzing ancestry data [32]. Lastly, the Native American/Alaska Native sample was classified into its own ethnic group, reflecting their distinctive genetic composition and underscoring the need to include this ethnic group in genetic studies [33]. These findings emphasize the importance of recognizing genetic diversity within ethnic groups when conducting genomic studies, as ancestral background can significantly influence genetic variability.

In cancer, FGA provides a crucial perspective on the genomic burden and tumor complexity. A high FGA may suggest greater genomic instability, which is often associated with increased aggressiveness and poorer prognosis across different cancer types, such as prostate cancer [34], lung adenocarcinoma [35], and non-small-cell lung cancer brain metastasis [36]. FGA analysis in BC samples reveals significant variations among different ethnic groups, reflecting how the burden of genomic alterations can differ by ethnicity. In this study, we observed significant differences in the median FGA between the White, Black or African American, Asian, and Hispanic or Latino groups, with lower median values observed in the White and Hispanic or Latino groups. In the White group, FGA values predominantly fall within the range of 0.05–0.25, suggesting moderate genomic alteration. In contrast, FGA values in the African American and Asian groups tend to be higher, ranging from 0.25 to 0.6, with no values exceeding 0.85. This trend suggests a generally higher overall burden of genomic alterations in these groups. For the Hispanic or Latino group, FGA values are also in the range of 0.05–0.25, potentially reflecting similar patterns to those observed in the White group. Finally, the Native American/Alaska Native sample shows a significantly lower FGA, with a value of 0.19.

These results show a wide range of genomic alteration burden in BC, suggesting that FGA profiles can differ significantly between groups. This variability underscores the need to adapt research and treatment approaches to address differences in genomic alterations and improve the precision of therapeutic strategies.

Statistically significant differences were observed in the mutation counts among ethnic groups. The Asian and Black or African American groups exhibited the highest median mutation counts, with values of 46 and 43, respectively. These values were notably higher compared to the White (37) and Hispanic or Latino groups (31). This suggests that ethnic background may play a role in the accumulation of mutations, which could be attributed to genetic, environmental, or lifestyle factors. Further investigation into the underlying causes of these differences could provide valuable insights into cancer genomics and the impact of ethnicity on mutation rates.

Before our gene filtering, we observed notable variability across different ethnic groups, with a significant diversity of mutated genes, including point mutations, SVs, and CNAs. In the White group, 14,796 genes had point mutations, 3831 genes had SVs, and 34,108 genes had CNAs. In the Black or African American group, 5886 genes had point mutations, 1564 genes had SVs, and 22,766 genes had CNAs. In the Asian group, 3441 genes had point mutations, 729 genes had SVs, and 1530 genes had CNAs. In the Hispanic or Latino group, 3849 genes had point mutations, 263 genes had SVs, and 7116 genes had CNAs. Finally, in the American Indian/Alaska Native group, 25 genes had point mutations, 28 genes had SVs, and 255 genes had CNAs. These findings highlight significant ethnic variability in mutation patterns, with certain groups displaying a higher frequency of mutations, especially in SVs and CNAs. This suggests that different ethnicities may experience distinct mutational landscapes, which could be influenced by genetic predispositions, environmental factors, or other underlying mechanisms.

When comparing the most mutated gene within each category (SVs, CNAs, and point mutations) across ethnic groups and analyzing its frequency in other ethnic groups, we observed distinctive patterns that suggest variations in genetic susceptibility and underlying tumor mechanisms. Previous studies have shown that certain genetic alterations may be influenced by ancestry, impacting both tumor biology and the response to specific treatments [37,38].

For SVs, a higher frequency of these mutations was observed in the Asian and Hispanic or Latino groups compared to the White and Black or African American groups.

*ERBB2* was significantly more frequent in the Asian group, which aligns with its higher activation in BC within this ethnic group and suggests a potential role of this oncogene’s amplification in tumor aggressiveness. This may help explain the higher incidence of Her2+ tumors in this group compared to other ethnicities [39]. In the Asian group, a higher frequency of mutations was observed in genes such as *FBXL20*, *STARD3*, *IKZF3*, *WIPF2*, and *ANKRD17*, with the latter being exclusive to this ethnic group. The mutation in *ANKRD17* is particularly significant, as its unique presence in this group suggests an ethnic-specific pattern in BC carcinogenesis. This underscores the importance of considering genetic diversity in mutation studies, as different ethnicities may exhibit mutational profiles that could influence cancer susceptibility and treatment outcomes. *ANKRD17*, a circular RNA, has been shown to promote cell growth, migration, and invasion in BC by regulating key molecules involved in cancer, such as certain micro-RNAs, primarily miR-143 [40].

On the other hand, *FBXL20* has been linked to chemotherapy resistance in cancer [41], while the suppression of *STARD3* expression induces cell death in BC, and its high expression is associated with poorer survival [42].

Additionally, *IKZF3* overexpression correlates with *ERBB2* in BC, suggesting its potential as a therapeutic target in Her2+ patients [43]. These findings highlight the importance of targeting specific molecular pathways in BC therapy, emphasizing the need for personalized treatments based on genetic and molecular profiles.

In the Hispanic or Latino group, SVs in *TSHZ2*, *FBXO47*, *ZMYND8*, and *RNF169* were more common, which could be related to differences in genomic stability. *TSHZ2* and *FBXO47* have been implicated in the regulation of the cell cycle [44,45] by controlling key molecules involved in this process, and their alteration could contribute to tumor progression. Meanwhile, *ZMYND8* plays a crucial role in cell cycle progression, invasion, and metastasis [46]. Additionally, the overexpression of *RNF169* has been linked to poor prognosis in pancreatic adenocarcinoma [47]. The higher frequency of alterations in these genes in Hispanic or Latino individuals may indicate a specific genetic predisposition that favors genomic instability and tumor aggressiveness in this group.

For CNAs, genes such as *PGAP3*, *MIEN1*, *ERBB2*, *IKZF3*, *GRB7*, *TCAP*, *PNMT*, *STARD3*, *PPP1R1B*, *MED2*4, *NEUROD2*, *ZPBP2*, and *CDK12* showed higher frequencies in the Asian group, reflecting a characteristic pattern of genomic instability frequently observed in this ethnic group. This elevated frequency of CNAs could indicate greater susceptibility to genomic alterations, potentially contributing to tumorigenesis and progression in the Asian ethnicity. In contrast, the White, Black or African American, and Hispanic or Latino groups presented lower frequencies of CNA in these genes. This discrepancy suggests that genomic instability may manifest differently across ethnicities, possibly due to genetic and environmental factors influencing tumor biology.

Previously, we described that genes such as *ERBB2*, *STARD3*, and *IKZF3* play a crucial role in cell proliferation and are primarily mutated in the Asian group, suggesting a tendency for these genes to mutate within this group. Furthermore, these alterations are frequently observed in this group in SV mutations, which reinforces the idea that genomic instability in this ethnicity may be associated with these specific genes.

On the other hand, other genes such as *PGAP3*, *MIEN1*, *GRB7*, *TCAP*, *PNMT*, *PPP1R1*B, *MED24*, *NEUROD2*, *ZPBP2*, and *CDK12* could also play an important role in BC. The overexpression of *PGAP3* and *MIEN1* has been linked to metastasis in BC patients [48,49], suggesting that they could be therapeutic targets for combating tumor spread. Additionally, the amplification of *GRB7*, *MED24*, and *NEUROD2* is correlated with *ERBB2* amplification [50,51,52], making them potential therapeutic targets in Her2+ patients. *CDK12* is recognized as a key regulator of the cell cycle, and it has been described as a marker of tumor progression in BC [53]. This finding suggests that *CDK12* could be a relevant therapeutic target for blocking tumor progression. However, the role of genes such as *PNMT*, *TCAP*, and *PPP1R1B* in BC and other cancers is still not well defined, requiring further research to understand their potential as biomarkers or therapeutic targets. The identification of mutations in these genes opens up new possibilities for targeted therapy and highlights the importance of understanding genetic variability between different ethnicities in cancer studies.

For point mutations, *TP53* was less frequent in the Hispanic or Latino group, suggesting that tumor development in this ethnic group could rely on alternative pathways to the classic disruption of the tumor suppressor *TP53*. Additionally, *PIK3CA* was less frequent in the Black or African American group, which could indicate the differential activation of key oncogenic pathways and have implications for responses to targeted therapies, as *PIK3CA* mutations are associated with sensitivity to PI3K inhibitors such as alpelisib [54].

The observed differences in mutations across ethnic groups highlight the need for personalized cancer treatments based on the specific genetic variations in each ethnicity. It is important to note that although some genes showed higher frequencies of SVs, CNAs, and point mutations in certain ethnic groups, as shown in Figure 4 and Figure 5, the overall frequencies of these alterations were not statistically different between the ethnicities. Therefore, these mutations should not be considered specific to any one ethnic group, but rather, they appear to be shared across the studied groups. However, the genes presented in Table 4 showed statistically significant differences among ethnic groups, suggesting that certain alterations may be influenced by genetic ancestry.

After applying our filtering criteria, we categorized the samples into “altered” and “non-altered” groups. The “altered” group contained mutations in the filtered genes, while the “non-altered” group had mutations in genes that escaped our filtering due to their low frequency in these groups.

In the White group, statistically significant differences were observed in the distribution of tumor type and subtype between the altered and non-altered groups. Notably, genes like *PIK3CA* showed a high frequency of mutations, and amplifications in genes such as *LTO1*, *ANO1*, and *CCND1* contributed to the complexity of BC. In the Black or African American group, significant differences were observed in tumor subtype between the altered and non-altered groups, with frequent mutations in *TP53* and structural variants in *BCAS3*. Additionally, amplifications in *MYC* were found, which could play a role in the observed variability. For the Asian group, significant differences were detected in both tumor type and subtype between the altered and non-altered groups. Mutations in *TP53*, SNVs in *FBXL*, and amplifications in genes like *PGAP3*, *MIEN1*, and *ERBB2* could contribute to this variability. In the Hispanic or Latino group, although no significant differences were found in clinical parameters, mutations in *TTN* and structural variants in *RARA* reflected a diverse mutational profile. However, these mutations did not translate into differences in tumor type or subtype.

These findings highlight the complex and heterogeneous nature of BC across different ethnic groups, where the impact of genetic alterations varies significantly. These results underscore the importance of considering ethnic-specific genetic factors in cancer research, as they may contribute to varying tumor behaviors and responses to treatment across ethnicities.

After filtering the genes in each ethnic group, we created a dataset by combining all the genes from the studied ethnic groups. A total of 71 genes were identified, each exhibiting various types of mutations including point mutations, SVs, and CNAs. This diverse range of genetic alterations highlights the complexity and multifaceted nature of BC genomics. Most frequently mutated genes are classified as kinases and enzymes. These genes play crucial roles in various signaling pathways that regulate cell growth, division, and survival. Mutations in these genes can lead to dysregulated signaling, contributing to tumor development and progression [55,56]. This finding is consistent with the understanding that kinases and enzymes are key players in cancer biology, often serving as potential targets for targeted therapies. Following kinases and enzymes, oncogenes are the second most frequently mutated category. Oncogenes, can drive cancer progression by promoting uncontrolled cell proliferation and inhibiting apoptosis [57]. The prominence of oncogenes among mutated genes underscores their central role in BC pathogenesis and emphasizes the importance of targeting these genes in therapeutic strategies. The third category, labeled “others”, includes a variety of gene types such as non-coding RNAs, pseudogenes, secreted and transmembrane proteins, components of ubiquitin ligases, and apoptosis regulators. This category reflects the broader genetic landscape of BC, where alterations in non-coding RNAs and other regulatory components can also contribute to tumorigenesis. For instance, non-coding RNAs have been increasingly recognized for their role in gene regulation and their potential as biomarkers for cancer [58,59,60]. Overall, the classification of mutated genes in BC reveals a broad spectrum of genetic alterations, with kinases, enzymes, and oncogenes being the most frequently affected categories. Understanding these patterns provides valuable insights into the molecular mechanisms underlying BC and highlights the need for comprehensive approaches in both research and clinical practice to address the diverse genetic alterations present in this disease.

The results obtained from the CancerHallmarks platform [21] analysis provide a comprehensive understanding of how the filtered and mutated genes in our ethnic BC groups are associated with distinct key biological processes. A significant finding is the identification of 24 genes related to “Sustaining Proliferative Signaling”, including prominent ones such as *TP53*, *PRKCA*, *ERBB2*, *MYC*, *CCND1*, and *PIK3CA*, underscoring that uncontrolled proliferation is one of the fundamental drivers of tumor growth [61,62]. Other equally relevant processes include “Replicative Immortality” and “Genome Instability”, each involving 10 genes, which allow cancer cells to evade cell death and continue accumulating mutations, becoming more aggressive over time. Genes like *TP53*, *CDK12*, and *MYC* play a key role in these processes [63,64,65].

One notable aspect is that the hallmark enrichment analyses show strong statistical significance in “Replicative Immortality” (*p* < 0.0001) and “Sustained Angiogenesis” (*p* = 0.00040) within the “Core Cancer Hallmark Gene Set”, reaffirming their relevance as potential therapeutic targets. Angiogenesis, facilitated by genes such as *ERBB2* [66,67] and *CCND1* [68,69], is crucial for supplying nutrients and oxygen to growing tumors [70,71], and this analysis suggests that its role could be even more important in certain BC subtypes.

Additionally, the significant association of 20 genes with “Evading Growth Suppressors” (*p* = 0.00144) and 15 genes with “Resisting Cell Death” (*p* = 0.00184) highlights cancer’s ability to reprogram cellular mechanisms, evading both apoptosis and growth-inhibitory signals. Genes like *CDH1*, *MAP3K1*, and *FADD* reflect these evasion mechanisms, which may explain the resistance of certain tumors to conventional therapies [61,72,73]. On the other hand, “Tissue Invasion and Metastasis”, involving 18 genes such as *MUC16*, *FGF4*, and *MAP3K1*, remains one of the leading causes of mortality in BC patients, emphasizing the importance of these genes in the tumor’s ability to spread to other tissues [74,75,76]. Furthermore, there is evidence of reprogrammed energy metabolism with eight genes involved, suggesting that cancer cells can metabolically adapt to thrive in unfavorable environments [77].

The analysis using the MSigDB “Hallmark Gene Sets” [22] and NDEx [23] through Cytoscape.org [24] revealed a detailed classification of genes that were initially not associated with any of the classic cancer hallmarks but, after evaluation, show clear involvement in several key processes. For example, genes such as *SYNE1* and *STARD3* were classified in the UV radiation response pathway, suggesting their relation to the hallmark of “Genome Instability and Mutation”, one of the pillars of tumor development [61,78,79]. This classification reinforces the fact that genomic instability is an important mechanism in the accumulation of mutations in cancer cells.

Other genes, such as *RYR2* and *ANO1*, both related to KRAS Signaling in the MSigDB “Hallmark Gene Sets” [22], were proposed for the hallmark of “Sustaining Proliferative Signaling”, underscoring their role in uncontrolled cell proliferation, a hallmark of cancer [61,62]. Likewise, the *TTN* gene, identified in the mitotic spindle pathway, was proposed for “Enabling Replicative Immortality”, reflecting its contribution to the cancer cells’ ability to proliferate indefinitely [80,81].

Based on the results obtained from the NDEx analysis [23] through Cytoscape.org [24], genes such as *PIP4K2B* were assigned to metabolic reprogramming, an emerging hallmark that highlights how cancer cells adapt their metabolism to support tumor growth [77]. These genes, along with others like *BPTF*, suggest that alterations in cellular metabolism are a key feature of BC, enabling malignant cells to survive in adverse environments.

Additionally, the *ZMYND8* gene, associated with the integrated BC pathway, was classified under the hallmark of “Sustaining Proliferative Signaling”, while *BCAS3*, related to ectoderm differentiation, was linked to “Tumor Promoting Inflammation” reinforcing the importance of inflammation in promoting tumor growth. Finally, *MSI2*, identified in the hepatitis C pathways, was proposed for the hallmarks of “Metabolic Reprogramming”, suggesting that these genes could be modulating the use of energy resources.

This combined analysis provides a broader framework for understanding how these genes, initially not associated with any cancer hallmark, may play crucial roles in tumor biology, opening up new avenues for research and the development of more targeted therapeutic strategies. However, it is important to clarify that the function or role in the cancer biology of many genes, such as *LINC00977*, *LINC02912*, *ASAP1-IT2*, *PGAP3*, *TCAP*, *ZPBP2*, *PNMT*, *BCAS3*, *ZMYND8*, *CCAT1*, *STARD3*, *PIP4K2B*, *FAM72C*, *TSHZ2*, *SRGAP2D*, *LINC00536*, *USP32*, *NEUROD2*, *LINC02584*, *CASC8*, *TTN*, *ANO1*, *RYR2*, *TRPS1*, *PRNCR1*, *LTO1*, *MSI2*, *TOP6BL*, *CCAT2*, *MICU1*, *SYNE1*, *NSD3*, *BPTF*, *FBXL20*, *WIPF2*, *FBXO47*, *PVT1*, *TANC2*, *PKHD1L1*, and *DNAAF11*, remains unclear. This highlights the importance of investigating their potential role in BC.

## 5. Conclusions

Our analysis highlights key mutated genes across ethnic groups that are significantly associated with fundamental processes driving BC, including “Sustained Proliferative Signaling”, “Genomic Instability”, and “Replicative Immortality”. The identification of these genes linked to these processes underscores their potential as targets for more precise and effective therapies.

Moreover, we observed significant differences in the frequency of certain mutated genes across ethnic groups, emphasizing the relevance of genetic diversity in BC. These variations may influence tumor progression through distinct molecular mechanisms, reinforcing the need to integrate diverse genetic backgrounds into therapeutic development. Addressing these differences could contribute to more accurate and effective treatments, underscoring the importance of incorporating ethnic diversity in BC research.

While the role of some genes observed in this study as mutated in BC is not fully understood, understanding their implications is crucial for advancing tumor biology knowledge, developing targeted treatments, and identifying new therapeutic targets. This finding opens up an interesting avenue for research and highlights a gap in knowledge that should be addressed in future studies.

## Figures and Tables

**Table 1:** Tumor type and subtype distribution by ethnicity.

Ethnicity	Tumor Type	n	%	Tumor Subtype	n	%
White	Infiltrating Ductal Carcinoma	425	65.3	Luminal A	333	51.2
	Infiltrating Lobular Carcinoma	150	23.0	Luminal B	114	17.5
	Others	34	5.2	Basal	93	14.3
	Mixed Histology	21	3.2	N/A *	58	8.9
	Mucinous Carcinoma	13	2.0	Her2	32	4.9
	Metaplastic Carcinoma	5	0.8	Normal like	21	3.2
	Medullary Carcinoma	2	0.3			
	Breast Invasive Carcinoma	1	0.2			
Black or African American	Infiltrating Ductal Carcinoma	136	81.9	Luminal A	51	30.7
	Infiltrating Lobular Carcinoma	13	7.8	Basal	48	28.9
	Other	7	4.2	Luminal B	24	14.5
	Mixed Histology	4	2.4	N/A *	22	13.3
	Medullary Carcinoma	3	1.8	Her2	13	7.8
	Metaplastic Carcinoma	1	0.6	Normal like	8	4.8
	Infiltrating Carcinoma	1	0.6			
	Mucinous Carcinoma	1	0.6			
Asian	Infiltrating Ductal Carcinoma	44	77.2	Luminal A	18	31.6
	Infiltrating Lobular Carcinoma	8	14.0	Luminal B	15	26.3
	Other	2	3.5	Her2	15	26.3
	Mucinous Carcinoma	2	3.5	Basal	7	12.3
	Metaplastic Carcinoma	1	1.8	Normal	1	1.8
				N/A *	1	1.8
Hispanic or Latino	Infiltrating Ductal Carcinoma	19	57.6	Luminal A	15	45.5
	Infiltrating Lobular Carcinoma	9	27.3	N/A *	6	18.2
	Other	2	6.1	Normal	4	12.1
	Metaplastic Carcinoma	1	3.0	Luminal B	4	12.1
	Mucinous Carcinoma	1	3.0	Basal	3	9.1
	Medullary Carcinoma	1	3.0	Her2	1	3.0

Note: * N/A; data not available.

**Table 2:** Frequency of the most commonly mutated genes across ethnic groups and their comparative analysis.

Mutation Type	Gene *	White	Black or African American	Asian	Hispanic or Latino	*p*
SV	*ERBB2*	0.94	0.62	8.77	0	<0.0001
	*FBXL20*	1.72	1.23	8.77	1.23	0.0039
	*STARD3*	0.63	1.85	7.02	0	0.000343
	*IKZF3*	0.94	1.85	7.02	0	0.002723
	*WIPF2*	0.78	0	5.26	0	0.002789
	*ANKRD17*	0	0	5.26	0	<0.0001
	*TSHZ2*	0.63	0	0	6.06	0.00122
	*FBXO47*	0	0	1.75	6.06	<0.0001
	*ZMYND8*	0.16	0.62	0	6.06	<0.0001
	*RNF169*	0.31	0.62	1.75	6.06	0.000859
CNA	*PGAP3*	9.75	11.52	33.33	6.06	<0.0001
	*MIEN1*	9.60	11.52	33.33	6.06	<0.0001
	*ERBB2*	9.75	11,52	33.33	6.06	<0.0001
	*IKZF3*	9.29	11,52	33.33	6.06	<0.0001
	*GRB7*	9.75	11.52	33.33	6.06	<0.0001
	*TCAP*	9.60	11.52	31.58	6.06	<0.0001
	*PNMT*	9.60	11.52	31.58	6.06	<0.0001
	*STARD3*	9.60	11.52	31.58	6.06	<0. 0001
	*PPP1R1B*	9.60	11.52	31.58	6.06	<0. 0001
	*MED24*	7.74	9.70	29.82	6.06	<0.0001
	*NEUROD2*	9.29	11.52	29.82	6.06	<0.0001
	*ZPBP2*	8.98	10.30	29.82	6.06	<0.0001
	*CDK12*	8.98	11.52	29.82	6.06	<0.0001
Point mutations	*TP53*	29.22	43.21	49.12	21.21	0.000127
	*PIK3CA*	35	19.14	38.60	27.27	0.000926

* only mutated genes that showed statistically significant differences in their frequencies between ethnic groups are included in the table.

**Table 3:** Functional classification of mutated genes in BC.

Category	Number of Genes	Genes
Oncogenes	12	*PIK3CA*, *PVT1*, *MYC*, *FGF19*, *FGF3*, *FGF4*, *CCAT2*, *CASC8*, *CCAT1*, *ERBB2*, *FGFR3*, *CCND1*
Tumor Suppressors	6	*TP53*, *CDH1*, *ZMYND8*, *FBXL20*, *FBXO47*, *PRKCA*
Transcription Factors and Regulators	10	*GATA3*, *KMT2C*, *BPTF*, *TRPS1*, *MSI2*, *IKZF3*, *RARA*, *POU5F1B*, *NEUROD2*, *MED24*
Structural Proteins	11	*TTN*, *MUC16*, *USH2A*, *SYNE1*, *SHANK2*, *TANC2*, *WIPF2*, *ANKRD17*, *FAM72C*, *DNAAF11*, *ZPBP2*
Kinases and Enzymes	14	*PIK3CA*, *MAP3K1*, *CDK12*, *PRKCA*, *PIP4K2B*, *KMT2C*, *MICU1*, *TOP6BL*, *UVRAG*, *RNF169*, *PGAP3*, *LTO1*, *PPP1R1B*, *FBXL20*
Receptors and Ion Channels	5	*CDH1*, *MUC16*, *USH2A*, *PKHD1L1*, *ANO1*
Metabolic Regulators and Transporters	5	*MICU1*, *PIP4K2B*, *PITPNC1*, *STARD3*, *VMP1*
Signaling Molecules	3	*MAP3K1*, *RYR2*, *FADD*
Others	10	Long non-coding RNAs: *LINC02584*, *LINC00536*, *LINC00977*, *ASAP1-IT2*, *PRNCR1*, *LINC02912*
		Pseudogenes: *SRGAP2D*
		Ubiquitin ligase components: *FBXL20*, *FBXO47*
		Apoptosis regulator: *MIEN1*

**Table 4:** Classification of genes by cancer hallmarks.

Hallmark	Genes
Sustained Angiogenesis	*GATA3*, *PIK3CA*, *MYC*, *TP53*, *CCND1*, *PRKCA*, *ERBB2*
Tumor-Promoting Inflammation	*GATA3*, *PIK3CA*, *TP53*, *FADD*, *PRKCA*, *CDH1*
Genome Instability	*GATA3*, *KMT2C*, *UVRAG*, *MYC*, *TP53*, *MAP3K1*, *CCND1*, *FADD*, *RNF169*, *CDK12*
Sustaining Proliferative Signaling	*TP53*, *PRKCA*, *PITPNC1*, *ERBB2*, *IKZF3*, *SHANK2*, *FGF3*, *ANKRD17*, *MYC*, *FGF4*, *GRB7*, *PPP1R1B*, *RARA*, *CCND1*, *GATA3*, *PIK3CA*, *CDH1*, *FGF19*, *MAP3K1*, *FADD*, *MED24*, *PPFIA1*
Evading Immune Destruction	*TP53*, *PRKCA*, *MYC*, *GATA3*, *PIK3CA*, *IKZF3*, *FADD*
Replicative Immortality	*TP53*, *PRKCA*, *CDK12*, *ERBB2*, *MYC*, *CCND1*, *PIK3CA*, *POU5F1B*, *KMT2C*
Resisting Cell Death	*TP53*, *PRKCA*, *ERBB2*, *MYC*, *FGF4*, *CCND1*, *GATA3*, *MIEN1*, *PIK3CA*, *CDH1*, *FGF19*, *MAP3K1*, *IKZF3*, *FADD*, *FGF3*
Evading Growth Suppressors	*TP53*, *PRKCA*, *PITPNC1*, *CDK12*, *ERBB2*, *KMT2C*, *FGF3*, *MYC*, *FGF4*, *GRB7*, *PPP1R1B*, *RARA*, *CCND1*, *GATA3*, *PIK3CA*, *CDH1*, *FGF19*, *MAP3K1*, *FADD*, *PPFIA1*
Reprogramming Energy Metabolism	*TP53*, *CDK12*, *ERBB2*, *MYC*, *CCND1*, *GATA3*, *PIK3CA*, *CDH1*
Tissue Invasion and Metastasis	*TP53*, *PRKCA*, *ERBB2*, *MUC16*, *KMT2C*, *FGF3*, *MYC*, *FGF4*, *GRB7*, *VMP1*, *USH2A*, *CCND1*, *GATA3*, *PIK3CA*, *CDH1*, *FGF19*, *MAP3K1*, *FADD*
Genes Not Linked to Any Hallmark	*LINC00977*, *LINC02912*, *ASAP1-IT2*, *PGAP3*, *TCAP*, *ZPBP2*, *PNMT*, *BCAS3*, *ZMYND8*, *CCAT1*, *STARD3*, *PIP4K2B*, *FAM72C*, *TSHZ2*, *SRGAP2D*, *LINC00536*, *USP32*, *NEUROD2*, *LINC02584*, *CASC8*, *TTN*, *ANO1*, *RYR2*, *TRPS1*, *PRNCR1*, *LTO1*, *MSI2*, *TOP6BL*, *CCAT2*, *MICU1*, *SYNE1*, *NSD3*, *BPTF*, *FBXL20*, *WIPF2*, *FBXO47*, *PVT1*, *TANC2*, *PKHD1L1*, *DNAAF11*

**Table 5:** Gene assignments based on the MSigDB Hallmark Gene Sets [22] and proposed cancer hallmarks.

Gene	MSigDB Classification	Proposed Hallmark
*SYNE1*	Hallmark UV Response DN	Genome Instability and Mutation
*RYR2*	Hallmark KRAS Signaling DN	Sustaining Proliferative Signaling
*ANO1*	Hallmark KRAS Signaling UP	Sustaining Proliferative Signaling
*TTN*	Hallmark Mitotic Spindle	Enabling Replicative Immortality
*STARD3*	Hallmark UV Response UP	Genome Instability and Mutation
*PVT1*	Hallmark P53 Pathway	Evading Growth Suppressors

**Table 6:** Gene assignments based on the NDEx [23] analysis on Cytoscape.org [24] and the proposed cancer hallmarks.

Gene	Molecular Pathway	Proposed Hallmark
*PIP4K2B*	Phosphoinositides Metabolism	Metabolic Reprogramming
*BPTF*	16p11.2 Proximal Deletion Syndrome	Altered Cellular Metabolism
*ZMYND8*	Integrated Breast Cancer Pathway	Sustaining Proliferative Signaling
*BCAS3*	Ectoderm Differentiation	Tumor Promoting Inflammation
*MSI2*	Hepatitis C-Human	Metabolic Reprogramming
*PIP4K2B*	Regulation Of Actin Cytoskeleton	Metabolic Reprogramming

## Data Availability

Data are contained within the article.

## Supplementary Materials

The following supporting information can be downloaded at: https://www.mdpi.com/article/10.3390/diseases13030086/s1, Table S1: Distribution of FGA and mutation count by ethnicity; Table S2: Statistical summary and *p*-values for FGA and mutation count across ethnic groups; Table S3: Top genes with the highest number of SVs by ethnicity; Table S4: Top genes with CNAs by ethnicity.

## References

[1] Hoxha I., Sadiku F., Hoxha L., Nasim M., Buteau M.A.C., Grezda K., Chamberlin M.D. (2024). Breast Cancer and Lifestyle Factors: Umbrella Review. Hematol./Oncol. Clin..

[2] Obeagu E.I., Obeagu G.U. (2024). Breast Cancer: A Review of Risk Factors and Diagnosis. Medicine.

[3] Xu Y., Gong M., Wang Y., Yang Y., Liu S., Zeng Q. (2023). Global Trends and Forecasts of Breast Cancer Incidence and Deaths. Sci. Data.

[4] Nguyen Hoang V.-A., Nguyen S.T., Nguyen T.V., Pham T.H., Doan P.L., Nguyen Thi N.T., Nguyen M.L., Dinh T.C., Pham D.H., Nguyen N.M. (2023). Genetic Landscape and Personalized Tracking of Tumor Mutations in Vietnamese Women with Breast Cancer. Mol. Oncol..

[5] Wilcox N., Dumont M., González-Neira A., Carvalho S., Joly Beauparlant C., Crotti M., Luccarini C., Soucy P., Dubois S., Nuñez-Torres R. (2023). Exome Sequencing Identifies Breast Cancer Susceptibility Genes and Defines the Contribution of Coding Variants to Breast Cancer Risk. Nat. Genet..

[6] Rooney M.M., Miller K.N., Plichta J.K. (2023). Genetics of Breast Cancer: Risk Models, Who to Test, and Management Options. Surg. Clin. North. Am..

[7] Ortiz M.M.O., Andrechek E.R. (2023). Molecular Characterization and Landscape of Breast Cancer Models from a Multi-Omics Perspective. J. Mammary Gland. Biol. Neoplasia.

[8] Miyashita M., Bell J.S.K., Wenric S., Karaesmen E., Rhead B., Kase M., Kaneva K., De La Vega F.M., Zheng Y., Yoshimatsu T.F. (2023). Molecular Profiling of a Real-World Breast Cancer Cohort with Genetically Inferred Ancestries Reveals Actionable Tumor Biology Differences between European Ancestry and African Ancestry Patient Populations. Breast Cancer Res..

[9] Nolan E., Lindeman G.J., Visvader J.E. (2023). Deciphering Breast Cancer: From Biology to the Clinic. Cell.

[10] Lakeman I.M.M., Rodríguez-Girondo M.D.M., Lee A., Celosse N., Braspenning M.E., van Engelen K., van de Beek I., van der Hout A.H., García E.B.G., Mensenkamp A.R. (2023). Clinical Applicability of the Polygenic Risk Score for Breast Cancer Risk Prediction in Familial Cases. J. Med. Genet..

[11] Makhnoon S., Levin B., Ensinger M., Mattie K., Volk R.J., Zhao Z., Mendoza T., Shete S., Samiian L., Grana G. (2023). A Multicenter Study of Clinical Impact of Variant of Uncertain Significance Reclassification in Breast, Ovarian and Colorectal Cancer Susceptibility Genes. Cancer Med..

[12] Guo L., Kong D., Liu J., Zhan L., Luo L., Zheng W., Zheng Q., Chen C., Sun S. (2023). Breast Cancer Heterogeneity and Its Implication in Personalized Precision Therapy. Exp. Hematol. Oncol..

[13] Johnson J.A., Moore B.J., Syrnioti G., Eden C.M., Wright D., Newman L.A. (2023). Landmark Series: The Cancer Genome Atlas and the Study of Breast Cancer Disparities. Ann. Surg. Oncol..

[14] Ding Y.C., Song H., Adamson A.W., Schmolze D., Hu D., Huntsman S., Steele L., Patrick C.S., Tao S., Hernandez N. (2023). Profiling the Somatic Mutational Landscape of Breast Tumors from Hispanic/Latina Women Reveals Conserved and Unique Characteristics. Cancer Res..

[15] Ullah A., Khan J., Yasinzai A.Q.K., Tracy K., Nguyen T., Tareen B., Garcia A.A., Heneidi S., Segura S.E. (2023). Metaplastic Breast Carcinoma in U.S. Population: Racial Disparities, Survival Benefit of Adjuvant Chemoradiation and Future Personalized Treatment with Genomic Landscape. Cancers.

[16] Gao J., Aksoy B.A., Dogrusoz U., Dresdner G., Gross B., Sumer S.O., Sun Y., Jacobsen A., Sinha R., Larsson E. (2013). Integrative Analysis of Complex Cancer Genomics and Clinical Profiles Using the cBioPortal. Sci. Signal.

[17] The Cancer Genome Atlas Program (TCGA)—NCI. https://www.cancer.gov/ccg/research/genome-sequencing/tcga.

[18] Weinstein J.N., Collisson E.A., Mills G.B., Shaw K.R.M., Ozenberger B.A., Ellrott K., Shmulevich I., Sander C., Stuart J.M. (2013). The Cancer Genome Atlas Pan-Cancer Analysis Project. Nat. Genet..

[19] Hoadley K.A., Yau C., Hinoue T., Wolf D.M., Lazar A.J., Drill E., Shen R., Taylor A.M., Cherniack A.D., Thorsson V. (2018). Cell-of-Origin Patterns Dominate the Molecular Classification of 10,000 Tumors from 33 Types of Cancer. Cell.

[20] Stelzer G., Rosen N., Plaschkes I., Zimmerman S., Twik M., Fishilevich S., Stein T.I., Nudel R., Lieder I., Mazor Y. (2016). The GeneCards Suite: From Gene Data Mining to Disease Genome Sequence Analyses. Curr. Protoc. Bioinform..

[21] Menyhart O., Kothalawala W.J., Győrffy B. (2024). A Gene Set Enrichment Analysis for the Cancer Hallmarks. J. Pharm. Anal..

[22] Liberzon A., Birger C., Thorvaldsdóttir H., Ghandi M., Mesirov J.P., Tamayo P. (2015). The Molecular Signatures Database (MSigDB) Hallmark Gene Set Collection. Cell Syst..

[23] Pillich R.T., Chen J., Churas C., Liu S., Ono K., Otasek D., Pratt D. (2021). NDEx: Accessing Network Models and Streamlining Network Biology Workflows. Curr. Protoc..

[24] Shannon P., Markiel A., Ozier O., Baliga N.S., Wang J.T., Ramage D., Amin N., Schwikowski B., Ideker T. (2003). Cytoscape: A Software Environment for Integrated Models of Biomolecular Interaction Networks. Genome Res..

[25] Xu H., Xu B. (2023). Breast Cancer: Epidemiology, Risk Factors and Screening. Chin. J. Cancer Res..

[26] Hendrick R.E., Monticciolo D.L., Biggs K.W., Malak S.F. (2021). Age Distributions of Breast Cancer Diagnosis and Mortality by Race and Ethnicity in US Women. Cancer.

[27] Ellington T.D., Henley S.J., Wilson R.J., Miller J.W., Wu M., Richardson L.C. (2023). Trends in Breast Cancer Mortality by Race/Ethnicity, Age, and US Census Region, United States─1999-2020. Cancer.

[28] Hurson A.N., Ahearn T.U., Koka H., Jenkins B.D., Harris A.R., Roberts S., Fan S., Franklin J., Butera G., Keeman R. (2024). Risk Factors for Breast Cancer Subtypes by Race and Ethnicity: A Scoping Review. JNCI J. Natl. Cancer Inst..

[29] Price A.L., Butler J., Patterson N., Capelli C., Pascali V.L., Scarnicci F., Ruiz-Linares A., Groop L., Saetta A.A., Korkolopoulou P. (2008). Discerning the Ancestry of European Americans in Genetic Association Studies. PLOS Genet..

[30] Zakharia F., Basu A., Absher D., Assimes T.L., Go A.S., Hlatky M.A., Iribarren C., Knowles J.W., Li J., Narasimhan B. (2009). Characterizing the Admixed African Ancestry of African Americans. Genome Biol..

[31] Banda Y., Kvale M.N., Hoffmann T.J., Hesselson S.E., Ranatunga D., Tang H., Sabatti C., Croen L.A., Dispensa B.P., Henderson M. (2015). Characterizing Race/Ethnicity and Genetic Ancestry for 100,000 Subjects in the Genetic Epidemiology Research on Adult Health and Aging (GERA) Cohort. Genetics.

[32] Bryc K., Durand E.Y., Macpherson J.M., Reich D., Mountain J.L. (2015). The Genetic Ancestry of African Americans, Latinos, and European Americans across the United States. Am. J. Hum. Genet..

[33] Bakhireva L.N., Nebeker C., Ossorio P., Angal J., Thomason M.E., Croff J.M. (2020). Inclusion of American Indians and Alaskan Natives in Large National Studies: Ethical Considerations and Implications for Biospecimen Collection in the HEALthy Brain and Child Development Study. Advers. Resil. Sci..

[34] Chakraborty G., Ghosh A., Nandakumar S., Armenia J., Mazzu Y.Z., Atiq M.O., Lee G.-S.M., Mucci L.A., Merghoub T., Wolchok J.D. (2020). Fraction Genome Altered (FGA) to Regulate Both Cell Autonomous and Non-Cell Autonomous Functions in Prostate Cancer and Its Effect on Prostate Cancer Aggressiveness. J. Clin. Oncol..

[35] Jones G.D., Brandt W.S., Shen R., Sanchez-Vega F., Tan K.S., Martin A., Zhou J., Berger M., Solit D.B., Schultz N. (2021). A Genomic-Pathologic Annotated Risk Model to Predict Recurrence in Early-Stage Lung Adenocarcinoma. JAMA Surg..

[36] Skakodub A., Walch H., Tringale K.R., Eichholz J., Imber B.S., Vasudevan H.N., Li B.T., Moss N.S., Hei Yu K.K., Mueller B.A. (2023). Genomic Analysis and Clinical Correlations of Non-Small Cell Lung Cancer Brain Metastasis. Nat. Commun..

[37] Tang W., Zhang F., Byun J.S., Dorsey T.H., Yfantis H.G., Ajao A., Liu H., Pichardo M.S., Pichardo C.M., Harris A.R. (2023). Population-Specific Mutation Patterns in Breast Tumors from African American, European American, and Kenyan Patients. Cancer Res. Commun..

[38] Galappaththi S.P.L., Smith K.R., Alsatari E.S., Hunter R., Dyess D.L., Turbat-Herrera E.A., Dasgupta S. (2024). The Genomic and Biologic Landscapes of Breast Cancer and Racial Differences. Int. J. Mol. Sci..

[39] Yu A.Y.L., Thomas S.M., DiLalla G.D., Greenup R.A., Hwang E.S., Hyslop T., Menendez C.S., Plichta J.K., Tolnitch L.A., Fayanju O.M. (2022). Disease Characteristics and Mortality among Asian Women with Breast Cancer. Cancer.

[40] Chen H., Zhang L.-F., Zhang L., Miao Y., Xi Y., Liu M.-F., Zhang M., Li B. (2023). CircANKRD17 Promotes Glycolysis by Inhibiting miR-143 in Breast Cancer Cells. J. Cell Physiol..

[41] Manne R.K., Agrawal Y., Malonia S.K., Banday S., Edachery S., Patel A., Kumar A., Shetty P., Santra M.K. (2021). FBXL20 Promotes Breast Cancer Malignancy by Inhibiting Apoptosis through Degradation of PUMA and BAX. J. Biol. Chem..

[42] Li P., Zhang Z., Lv H., Sun P. (2022). Inhibiting the Expression of STARD3 Induced Apoptosis via the Inactivation of PI3K/AKT/mTOR Pathway on ER+ Breast Cancer. Tissue Cell.

[43] Lin C.-Y., Yu C.-J., Shen C.-I., Liu C.-Y., Chao T.-C., Huang C.-C., Tseng L.-M., Lai J.-I. (2022). IKZF3 Amplification Frequently Occurs in HER2-Positive Breast Cancer and Is a Potential Therapeutic Target. Med. Oncol..

[44] Ma A., Yang Y., Cao L., Chen L., Zhang J.V. (2024). FBXO47 Regulates Centromere Pairing as Key Component of Centromeric SCF E3 Ligase in Mouse Spermatocytes. Commun. Biol..

[45] Uribe M.L., Dahlhoff M., Batra R.N., Nataraj N.B., Haga Y., Drago-Garcia D., Marrocco I., Sekar A., Ghosh S., Vaknin I. (2021). TSHZ2 Is an EGF-Regulated Tumor Suppressor That Binds to the Cytokinesis Regulator PRC1 and Inhibits Metastasis. Sci. Signal.

[46] Chen Y., Tsai Y.-H., Tseng S.-H. (2021). Regulation of ZMYND8 to Treat Cancer. Molecules.

[47] Wang J., Chen H., Deng Q., Chen Y., Wang Z., Yan Z., Wang Y., Tang H., Liang H., Jiang Y. (2023). High Expression of RNF169 Is Associated with Poor Prognosis in Pancreatic Adenocarcinoma by Regulating Tumour Immune Infiltration. Front. Genet..

[48] Hao N., Li M., Wang J., Song Y., Zhao Y., Zhang L., Yang X., Chen L., Ma J., Jia Q. (2023). High PGAP3 Expression Is Associated with Lymph Node Metastasis and Low CD8+T Cell in Patients with HER2+ Breast Cancer. Pathol. Res. Pract..

[49] Zhao H.-B., Zhang X.-F., Wang H.-B., Zhang M.-Z. (2017). Migration and Invasion Enhancer 1 (MIEN1) Is Overexpressed in Breast Cancer and Is a Potential New Therapeutic Molecular Target. Genet. Mol. Res..

[50] Bivin W.W., Yergiyev O., Bunker M.L., Silverman J.F., Krishnamurti U. (2017). GRB7 Expression and Correlation With HER2 Amplification in Invasive Breast Carcinoma. Appl. Immunohistochem. Mol. Morphol..

[51] van den Ende N.S., Smid M., Timmermans A., van Brakel J.B., Hansum T., Foekens R., Trapman A.M.A.C., Heemskerk-Gerritsen B.A.M., Jager A., Martens J.W.M. (2022). HER2-Low Breast Cancer Shows a Lower Immune Response Compared to HER2-Negative Cases. Sci. Rep..

[52] Lacle M.M., Moelans C.B., Kornegoor R., van der Pol C., Witkamp A.J., van der Wall E., Rueschoff J., Buerger H., van Diest P.J. (2015). Chromosome 17 Copy Number Changes in Male Breast Cancer. Cell Oncol..

[53] Peng F., Yang C., Kong Y., Huang X., Chen Y., Zhou Y., Xie X., Liu P. (2020). CDK12 Promotes Breast Cancer Progression and Maintains Stemness by Activating C-Myc/β-Catenin Signaling. Curr. Cancer Drug Targets.

[54] André F., Ciruelos E., Rubovszky G., Campone M., Loibl S., Rugo H.S., Iwata H., Conte P., Mayer I.A., Kaufman B. (2019). Alpelisib for PIK3CA-Mutated, Hormone Receptor-Positive Advanced Breast Cancer. N. Engl. J. Med..

[55] Cicenas J., Zalyte E., Bairoch A., Gaudet P. (2018). Kinases and Cancer. Cancers.

[56] Singha M., Pu L., Srivastava G., Ni X., Stanfield B.A., Uche I.K., Rider P.J.F., Kousoulas K.G., Ramanujam J., Brylinski M. (2023). Unlocking the Potential of Kinase Targets in Cancer: Insights from CancerOmicsNet, an AI-Driven Approach to Drug Response Prediction in Cancer. Cancers.

[57] Kontomanolis E.N., Koutras A., Syllaios A., Schizas D., Mastoraki A., Garmpis N., Diakosavvas M., Angelou K., Tsatsaris G., Pagkalos A. (2020). Role of Oncogenes and Tumor-Suppressor Genes in Carcinogenesis: A Review. Anticancer Res..

[58] Tan Y.-T., Lin J.-F., Li T., Li J.-J., Xu R.-H., Ju H.-Q. (2021). LncRNA-Mediated Posttranslational Modifications and Reprogramming of Energy Metabolism in Cancer. Cancer Commun..

[59] Jin H., Du W., Huang W., Yan J., Tang Q., Chen Y., Zou Z. (2021). lncRNA and Breast Cancer: Progress from Identifying Mechanisms to Challenges and Opportunities of Clinical Treatment. Mol. Ther. Nucleic Acids.

[60] Dvorská D., Braný D., Ňachajová M., Halašová E., Danková Z. (2021). Breast Cancer and the Other Non-Coding RNAs. Int. J. Mol. Sci..

[61] Hanahan D. (2022). Hallmarks of Cancer: New Dimensions. Cancer Discov..

[62] Feitelson M.A., Arzumanyan A., Kulathinal R.J., Blain S.W., Holcombe R.F., Mahajna J., Marino M., Martinez-Chantar M.L., Nawroth R., Sanchez-Garcia I. (2015). Sustained Proliferation in Cancer: Mechanisms and Novel Therapeutic Targets. Semin. Cancer Biol..

[63] Blondeaux E., Arecco L., Punie K., Graffeo R., Toss A., De Angelis C., Trevisan L., Buzzatti G., Linn S.C., Dubsky P. (2023). Germline *TP53* Pathogenic Variants and Breast Cancer: A Narrative Review. Cancer Treat. Rev..

[64] Papadimitropoulou A., Makri M., Zoidis G. (2024). MYC the Oncogene from Hell: Novel Opportunities for Cancer Therapy. Eur. J. Med. Chem..

[65] Lui G.Y.L., Grandori C., Kemp C.J. (2018). CDK12: An Emerging Therapeutic Target for Cancer. J. Clin. Pathol..

[66] Li F., Meng G., Tan B., Chen Z., Ji Q., Wang X., Liu C., Niu S., Li Y., Liu Y. (2021). Relationship between HER2 Expression and Tumor Interstitial Angiogenesis in Primary Gastric Cancer and Its Effect on Prognosis. Pathol. Res. Pract..

[67] Ciesielski M., Szajewski M., Pęksa R., Lewandowska M.A., Zieliński J., Walczak J., Szefel J., Kruszewski W.J. (2018). The Relationship between HER2 Overexpression and Angiogenesis in Gastric Cancer. Medicine.

[68] Hussen B.M., Hidayat H.J., Ghafouri-Fard S. (2022). Identification of Expression of CCND1-Related lncRNAs in Breast Cancer. Pathol. Res. Pract..

[69] Pi J., Liu J., Zhuang T., Zhang L., Sun H., Chen X., Zhao Q., Kuang Y., Peng S., Zhou X. (2018). Elevated Expression of miR302-367 in Endothelial Cells Inhibits Developmental Angiogenesis via CDC42/CCND1 Mediated Signaling Pathways. Theranostics.

[70] Liu Z.-L., Chen H.-H., Zheng L.-L., Sun L.-P., Shi L. (2023). Angiogenic Signaling Pathways and Anti-Angiogenic Therapy for Cancer. Signal Transduct. Target. Ther..

[71] Mou J., Li C., Zheng Q., Meng X., Tang H. (2024). Research Progress in Tumor Angiogenesis and Drug Resistance in Breast Cancer. Cancer Biol. Med..

[72] D’Amico M., De Amicis F. (2024). Challenges of Regulated Cell Death: Implications for Therapy Resistance in Cancer. Cells.

[73] Luque-Bolivar A., Pérez-Mora E., Villegas V.E., Rondón-Lagos M. (2020). Resistance and Overcoming Resistance in Breast Cancer. Breast Cancer Targets Ther..

[74] Jiang W.G., Sanders A.J., Katoh M., Ungefroren H., Gieseler F., Prince M., Thompson S.K., Zollo M., Spano D., Dhawan P. (2015). Tissue Invasion and Metastasis: Molecular, Biological and Clinical Perspectives. Semin. Cancer Biol..

[75] Fares J., Fares M.Y., Khachfe H.H., Salhab H.A., Fares Y. (2020). Molecular Principles of Metastasis: A Hallmark of Cancer Revisited. Signal Transduct. Target. Ther..

[76] Liu M., Yang J., Xu B., Zhang X. (2021). Tumor Metastasis: Mechanistic Insights and Therapeutic Interventions. MedComm.

[77] Nong S., Han X., Xiang Y., Qian Y., Wei Y., Zhang T., Tian K., Shen K., Yang J., Ma X. (2023). Metabolic Reprogramming in Cancer: Mechanisms and Therapeutics. MedComm.

[78] Sonugür F.G., Akbulut H. (2019). The Role of Tumor Microenvironment in Genomic Instability of Malignant Tumors. Front. Genet..

[79] Andor N., Maley C.C., Ji H.P. (2017). Genomic Instability in Cancer: Teetering on the Limit of Tolerance. Cancer Res..

[80] Han X., Chen J., Wang J., Xu J., Liu Y. (2022). TTN Mutations Predict a Poor Prognosis in Patients with Thyroid Cancer. Biosci. Rep..

[81] Pease J.C., Tirnauer J.S. (2011). Mitotic Spindle Misorientation in Cancer—Out of Alignment and into the Fire. J. Cell Sci..

