# Supplementary material for: Genomic Landscape of Breast Cancer: Study Across Diverse Ethnic Groups
Source: Diseases. 2025 Mar 17;13(3):86. doi: 10.3390/diseases13030086 (PMC11941751; doi:10.3390/diseases13030086)
Supplement: Supplementary file 1 [file diseases-13-00086-s001.zip › diseases-3480122-supplementary.pdf]

Table S1. Distribution of FGA and mutation count by ethnicity.

Ethnicity	FGA	N (FGA)	FGA Proportion	Mutation Count	N (Mutation Count)	Mutation Cunt Proportion
**White**	<0.05	70	0.1084	<10	10	0.0165
**White**	0.05-0.25	275	0.4276	10-30	227	0.3748
**White**	0.25-0.6	240	0.3721	30-50	147	0.2426
**White**	0.6-0.85	49	0.0761	50-70	84	0.1385
**White**	0.85-120	10	0.0155	70-90	43	0.0711
**White**	>120	62	0.0961	90-120	32	0.0528
				>120	62	0.1025
**Total White**		**644**	**1**		**605**	**1**
**Black or African American**	<0.05	10	0.0606	<10	2	0.0136
**Black or African American**	0.05-0.25	57	0.3455	10-30	39	0.2653
**Black or African American**	0.25-0.6	85	0.5152	30-50	45	0.3061
**Black or African American**	0.6-0.85	13	0.0788	50-70	21	0.1429
**Black or African American**	0.85-120	0	0	70-90	13	0.0884
**Black or African American**	>120	0	0	90-120	10	0.068
**Black or African American**				>120	17	0.1156
**Total Black or African American**	**165**	**1**		**147**	**1**	
**Asian**	<0.05	5	0.0735	<10	0	0
**Asian**	0.05-0.25	20	0.2941	10-30	12	0.2143
**Asian**	0.25-0.6	28	0.4118	10-50	18	0.3214
**Asian**	0.6-0.85	4	0.0588	50-70	10	0.1786
**Asian**	0.85-120	0	0	70-90	2	0.0357
**Asian**	>120	0	0	90-120	3	0.0536
**Asian**				>120	11	0.1964
**Total Asian**		**68**	**1**		**56**	**1**
**Hispanic or Latin**	<0.05	5	0.1515	<10	3	0.1034

**Hispanic or Latin**	0.05-0.25	16	0.4848	oct-30	11	0.3793
**Hispanic or Latin**	0.25-0.6	10	0.303	30-50	7	0.2414
**Hispanic or Latin**	0.6-0.85	1	0.0303	50-70	3	0.1034
**Hispanic or Latin**	0.85-120	1	0.0303	70-90	2	0.0689
**Hispanic or Latin**	>120	0	0	90-120	1	0.0345
**Hispanic or Latin**				>120	2	0.0689
**Total Hispanic or Latin**		**33**	**1**		**29**	**1**

FGA; Fraction of Genome Altered

Table S2. Statistical Summary and p-values for FGA and Mutation Count Across Ethnic Groups

Statistic	Asian	Black or African American	Hispanic or Latino	White
**FGA**				
**Count**	57	165	33	644
**Minimum**	0	0	0	0
**Maximum**	0.74	0.79	0.9	1
**Mean**	0.31	0.32	0.24	0.28
**Standard Deviation**	0.18	0.2	0.21	0.21
**Median**	0.3	0.32	0.19	0.24
**Mean Absolute Deviation**	0.12	0.16	0.1	0.14
**25% (Q1)**	0.18	0.15	0.09	0.12
**75% (Q3)**	0.44	0.47	0.33	0.42
**Mutation Count**				
**Count**	56	147	29	605
**Minimum**	16	4	1	3
**Maximum**	633	459	3879	5400
**Mean**	77.86	59.4	170.76	88.13
**Standard Deviation**	93.03	50.47	701.4	323.05
**Median**	46	43	31	37
**Mean Absolute Deviation**	18	16	13	16
**25% (Q1)**	34	29	19	25
**75% (Q3)**	82	74	51	66

Table S3. Top genes with the highest number of SVs by ethnicity

Ethnicity	Gene	Numer of SV*	Numer of samples**	Frequency	SV event
**White**	*SHANK2*	18	16	2.50	EMSY-SHANK2 Fusion
					PAK1-SHANK2 Fusion
					BCAS3-SHANK2 Fusion
					PIP4K2B-SHANK2 Fusion
					PLEKHA2-SHANK2 Fusion
					RAB6A-SHANK2 Fusion
					SHANK2-AHNAK Fusion
					SHANK2-ANXA13 Fusion
					SHANK2-KDM2A Fusion
					SHANK2-LILRB2 Fusion
					SHANK2-MYEOV Fusion
					SHANK2-MYEOV Fusion
					SHANK2-OPCML Fusion
					SHANK2-PGA5 Fusion
					SHANK2-PRKCA Fusion
					SHANK2-RBM4B Fusion
					SHANK2-SPTBN2 Fusion
					PAPD5-SHANK2 Fusion
	BCAS3	17	13	2%	BCAS3-RAD51C Fusion
					BCAS3-KAT7 Fusion
					IKZF3-BCAS3 Fusion
					PRKAR1A-BCAS3 Fusion
					ZNF217-BCAS3 Fusion
					BCAS3-ANLN Fusion
					BCAS3-APPBP2 Fusion
					BCAS3-CA4 Fusion
					BCAS3-CYTH1 Fusion
					BCAS3-DISP1 Fusion
					BCAS3-GH2 Fusion

					BCAS3-HEATR6 Fusion
					BCAS3-RNF169 Fusion
					BCAS3-SHANK2 Fusion
					BCAS3-TBC1D3P1 Fusion
					BCAS3-WIPI1 Fusion
					SKAP1-BCAS3 Fusion
	CDK12	13	12	1.90%	ATF4-CDK12 Fusion
					CDK12-IKZF3 Fusion
					CDK12-NUFIP2 Fusion
					PDK2-CDK12 Fusion
					PHF12-CDK12 Fusion
					RAP1GDS1-CDK12 Fusion
					RARA-CDK12 Fusion
					SSH2-CDK12 Fusion
					TIAM2-CDK12 Fusion
					TUBD1-CDK12 Fusion
					RARA-CDK12 Fusion
					C17orf98-CDK12 Fusion
					WIPF2-CDK12 Fusion
	NSD3	12	12	1.90%	IMPG1-NSD3 Fusion
					LAMTOR1-NSD3 Fusion
					LSM1-NSD3 Fusion
					NSD3-ANK1 Fusion
					NSD3-CSMD1 Fusion
					NSD3-GAB2 Fusion
					NSD3-PLAT Fusion
					NSD3-PLEKHA2 Fusion
					NSD3-PSD3 Fusion
					NSD3-RAB11FIP1 Fusion
					NSD3-SNX20 Fusion
					RNF122-NSD3 Fusion
**Black or African American**	*BCAS3*	8	6	3.7%	ABCA5-BCAS3 Fusion
					BCAS3-KRTAP4 Fusion
					BCAS3-EYA2 Fusion
					BCAS3-NFS1 Fusion
					BCAS3-RNFT1 Fusion
					BCAS3-VMP1 Fusion
					BCAS3-VMP1 Fusion
	CDK12	7	5	3.1%	ARSG-CDK12 Fusion
					CDK12-ANKRD13B Fusion

					CDK12-FAM20A Fusion
					CDK12-JAK2 Fusion
					CDK12-JUP Fusion
					CDK12-PLEKHM1P1 Fusion
					SAR1A-CDK12 Fusion
	MICU1	4	4	2.5%	ASCC1-MICU1 Fusion
					ASCC1-MICU1 Fusion
					MICU1-TSPAN3 Fusion
					TACC2-MICU1 Fusion
	PITPNC1	5	4	2.5%	FBXL20-PITPNC1 Fusion
					PIGU-PITPNC1 Fusion
					PITPNC1-ANXA10 Fusion
					PITPNC1-B4GALNT2 Fusion
					PITPNC1-BPTF Fusion
	UVRAG	4	4	2.5%	UVRAG-GDPD4 Fusion
					UVRAG-INTS4 Fusion
					UVRAG-MYEOV Fusion
					UVRAG-SHANK2 Fusion
	BPTF	4	4	2.5%	BPTF-ARSG Fusion
					BPTF-NOL11 Fusion
					BPTF-PLXDC1 Fusion
					PITPNC1-BPTF Fusion
	TRPS1	5	4	2.5%	TRPS1-LRP12 Fusion
					TRPS1-PAFAH1B1 Fusion
					TRPS1-C19orf66 Fusion
					TRPS1-SPAG1 Fusion
					TRPS1-ZNRF2 Fusion
	ANO1	4	4	2.5%	PC-ANO1 Fusion
					ANO1-EMSY Fusion
					RNF121-ANO1 Fusion
					THAP12-ANO1 Fusion
**Asian**	*FBXL20*	7	5	8.80%	CANT1-FBXL20 Fusion
					FBXL20-CANT1 Fusion
					FBXL20-CDC6 Fusion
					FBXL20-FBXO47 Fusion
					FBXL20-LRSAM1 Fusion
					FBXL20-WIPF2 Fusion

					NAGLU-FBXL20 Fusion
	ERBB2	5	5	8.80%	CORO1B-ERBB2 Fusion
					ERBB2-ABI3 Fusion
					ERBB2-PPP1R1B Fusion
					ERBB2-SDF4 Fusion
					MYO18A-ERBB2 Fusion
	STARD3	4	4	7%	STARD3-IKZF3 Fusion
					ABI3-STARD3 Fusion
					STARD3-CASC3 Fusion
					STARD3-STRADA Fusion
	MSI2	6	4	7%	MSI2-EFCAB5 Fusion
					MSI2-PPM1E Fusion
					MSI2-PRR15L Fusion
					MSI2-TRIM25 Fusion
					NR2F2-MSI2 Fusion
					SYNRG-MSI2 Fusion
	IKZF3	4	4	7%	RAD9A-IKZF3 Fusion
					RAPGEFL1-IKZF3 Fusion
					STARD3-IKZF3 Fusion
					WIPF2-IKZF3 Fusion
	TANC2	4	4	7%	TANC2-SSH2 Fusion
					TANC2-WFDC21P Fusion
					TEX2-TANC2 Fusion
					USP32-TANC2 Fusion
	SHANK2	4	4	7%	CREB3L2-SHANK2 Fusion
					NADSYN1-SHANK2 Fusion
					SHANK2-C7orf57 Fusion
					SHANK2-PATL1 Fusion
	WIPF2	4	4	7%	WIPF2-GRB7 Fusion
					WIPF2-IKZF3 Fusion
					FBXL20-WIPF2 Fusion
					WIPF2-IL2RB Fusion
	PIP4K2B	3	3	5.30%	PIP4K2B-MLLT6 Fusion
					BPTF-PIP4K2B Fusion
					PIP4K2B-KRT10-AS1 Fusion
	PRKCA	3	3	5.30%	INO80-PRKCA Fusion

					PEMT-PRKCA Fusion
					PSMD12-PRKCA Fusion
	ANKRD17	4	3	5.30%	ANKRD17-GC Fusion
					ANKRD17-KCNIP4 Fusion
					ANKRD17-RPRD1B Fusion
					RPRD1B-ANKRD17 Fusion
	TOP6BL	3	3	5.30%	SYT12-C11orf80 Fusion
					TMX2-C11orf80 Fusion
					C11orf80-PPP6R3 Fusion
	PVT1	3	3	5.30%	CASC11-PVT1 Fusion
					PVT1-PHF20L1 Fusion
					PVT1-SLC30A8 Fusion
	VMP1	3	3	5.30%	MED13-VMP1 Fusion
					PPM1E-VMP1 Fusion
					VMP1-CDC42EP4 Fusion
	USP32	3	3	5.30%	USP32-SCPEP1 Fusion
					USP32-TANC2 Fusion
					USP32-UBE2O Fusion
**Hispanic or Latin**	*ZMYND8*	2	2	6.10%	ZMYND8-C2orf27AP1 Fusion
					ZMYND8-GTPBP1 Fusion
	TSHZ2	2	2	6.10%	STK4-TSHZ2 Fusion
					TSTD3-TSHZ2 Fusion
	RNF169	2	2	6.10%	C2CD3-RNF169 Fusion
					RNF169-ANAPC15 Fusion
	RARA	3	2	6.10%	RARA-PSMD3 Fusion
					RARA-STAT3 Fusion
					TPCN2-RARA Fusion
	FBXO47	2	2	6.10%	CWC25-FBXO47 Fusion
					MED1-FBXO47 Fusion

SV: Structural Variant
*Total number of mutations
**Percentage of samples with one or more mutations

Table S4. Top Genes with CNA by ethnicity.

Ethnicity	Gene	Cytoband	Numer of samples**	Frequency	CNA
**White**	*LTO1*	11q13.2	93	14.40%	AMP
	ANO1	11q13.3	93	14.40%	AMP
	CCND1	11q13.3	93	14.40%	AMP
	LINC02584	11q13.3	92	14.20%	AMP
	FGF19	11q13.3	92	14.20%	AMP
	FGF3	11q13.3	91	14.10%	AMP
	FGF4	11q13.3	91	14.10%	AMP
	PPFIA1	11q13.3	91	14.10%	AMP
	FADD	11q13.3	91	14.10%	AMP
**Black or African American**	*MYC*	8q24.21	31	18.80%	AMP
	CCAT2	8q24.21	28	17%	AMP
	CASC8	8q24.21	28	17%	AMP
	POU5F1B	8q24.21	28	17%	AMP
	FAM72C	1q21.1	25	15.20%	AMP
	SRGAP2D	1q21.1	25	15.20%	AMP
	CCAT1	8q24.21	25	15.20%	AMP
	LINC00536	8q23.3	25	15.20%	AMP
**Asian**	*PGAP3*	17q12	19	33.30%	AMP
	MIEN1	17q12	19	33.30%	AMP
	ERBB2	17q12	19	33.30%	AMP
	IKZF3	17q12q-21.1	19	33.30%	AMP
	GRB7	17q12	19	33.30%	AMP
	TCAP	17q12	18	31.60%	AMP
	PNMT	17q12	18	31.60%	AMP
	STARD3	17q1211-q12	18	31.60%	AMP
	PPP1R1B	17q12	18	31.60%	AMP
	MED24	17q12.1	17	29.80%	AMP
	NEUROD2	17q12	17	29.80%	AMP
	ZPBP2	17q12.1	17	29.80%	AMP
	CDK12	17q12	17	29.80%	AMP
**Hispanic or Latin**	*PRNCR1*	8q24.21	6	18.20%	AMP
	LINC02912	8q24.21	6	18.20%	AMP

	LINC00977	8q24.21	6	18.20%	AMP
	ASAP1-IT2	8q24.22	6	18.20%	AMP
	DNAAF11	8q24.22	6	18.20%	AMP

AMP: Amplification.
